# Knowledge, Attitude, and Practice of Hand Hygiene among Medical and Nursing Students at a Tertiary Health Care Centre in Raichur, India

**DOI:** 10.1155/2014/608927

**Published:** 2014-02-06

**Authors:** Sreejith Sasidharan Nair, Ramesh Hanumantappa, Shashidhar Gurushantswamy Hiremath, Mohammed Asaduddin Siraj, Pooja Raghunath

**Affiliations:** ^1^Navodaya Medical College, Raichur, Karnataka 584103, India; ^2^Department of Community Medicine, Navodaya Medical College, Raichur, Karnataka 584103, India; ^3^Department of Microbiology, Pushpagiri Institute of Medical Science and Research Center, Tiruvalla, Kottayam, Kerala, India

## Abstract

*Background.* Hand hygiene is recognized as the leading measure to prevent cross-transmission of microorganisms. Regarding hospital acquired infections, the compliance of nurses with hand washing guidelines seems to be vital in preventing the disease transmission among patients. There is a paucity of studies exploring this subject in Asia. Especially medical and nursing student's knowledge of standard hand hygiene precautions is rarely compared. *Methods.* A cross-sectional study was conducted among 98 medical and 46 nursing students in a tertiary medical college in India. Knowledge was assessed using WHO hand hygiene questionnaire. Attitude and practices were evaluated by using another self-structured questionnaire. *Z* test was used to compare the percentage of correct responses between medical and nursing students. A *P* value less than 0.05 was considered significant. *Results.* Only 9% of participants (13 out of 144) had good knowledge regarding hand hygiene. Nursing students knowledge (*P* = 0.023) , attitude (*P* = 0.023), and practices (*P* < 0.05) were significantly better than medical students.

## 1. Introduction

Hand hygiene is recognized as the leading measure to prevent cross-transmission of microorganisms and to reduce the incidence of health care associated infections [1, 2]. Despite the relative simplicity of this procedure, compliance with hand hygiene among health care providers is as low as 40% [3–5]. To address this problem, continuous efforts are being made to identify effective and sustainable strategies. One of such efforts is the introduction of an evidence-based concept of “My five moments for hand hygiene” by World Health Organization. These five moments that call for the use of hand hygiene include the moment before touching a patient, before performing aseptic and clean procedures, after being at risk of exposure to body fluids, after touching a patient, and after touching patient surroundings. This concept has been aptly used to improve understanding, training, monitoring, and reporting hand hygiene among healthcare workers [6].

Nurses constitute the largest percentage of the health care workers (HCW) [7] and they are the “nucleus of the health care system” [8]. Because they spend more time with patients than any other HCWs, their compliance with hand washing guidelines seems to be more vital in preventing the disease transmission among patients.

In Asia there is a paucity of studies [7–10] exploring this subject, although the prevalence of health care associated infections is high in this region; especially medical and nursing student's knowledge of standard precautions is rarely compared [11]. The observance of hygiene by students is reported as being weak [12, 13]. Therefore, it is absolutely essential to investigate and know nurse's knowledge, attitudes, and practices about hand washing so that appropriate strategies can be developed to promote hand washing compliance.

## 2. Material and Method

This cross-sectional study was conducted in Navodaya Medical College (NMC), one of the biggest teaching hospitals in Raichur, India. This district is one of the 30 most backward places in India. NMC provides tertiary medical care for residents of Raichur and patients referred from neighboring states.

Ethical clearance was obtained from the Ethical Review Committee of Navodaya Medical College. Medical and nursing students were explained the content and nature of the study. Verbal consent was obtained from 98 medical and 46 nursing students who volunteered to participate. A self-administered questionnaire containing a set of questions regarding hand-hygiene knowledge, attitudes, and practices was distributed to all participants.

Knowledge was assessed using WHO's hand hygiene questionnaire for health care workers. This proforma of 25 questions includes multiple choice and “yes” or “no” questions. Attitude and practice were assessed using another self-structured questionnaire which consists of 10 and 6 questions, respectively. Respondents were given the option to select on a 1- to 7-point scale between strongly agree and strongly disagree. A score of 0 was given for negative attitudes and puny practices. 1 point was given for each correct response to positive attitudes and good practices so that maximum score for attitude is 10 and for practice it is 6. A score of more than 75% was considered good, 50–74% moderate, and less than 50% was taken as poor. Data was analyzed using SPSS version software. Descriptive statistics was used to calculate percentages for each of the responses given. *Z* test was used to compare the percentage of correct responses between medical and nursing students. A *P* value less than 0.05 was considered significant.

## 3. Results

There were a total of 144 study participants (46 nursing students and 98 medical students). In this a majority (79%, 114 out of 144) had claimed to have received formal training in hand washing. A significant difference (*P* < 0.001) was observed between medical (73 out of 98, (74.2%)) and nursing (44 out of 46, (95.4%)) students who had received formal training in hand hygiene. When asked about the correct technique of hand washing, 89 out of 98 medical students (91.3%) and 45 of 46 nursing students (97.8%) said they knew the correct technique of hand washing.

## 4. Knowledge on Hand Hygiene

The knowledge on hand hygiene was moderate (107 out of 144, 74%) among the total study population. Only 9% of participants (13 out of 144) had good knowledge regarding hand hygiene. Nursing students had significantly better knowledge than medical students. (*P* = 0.023) The percentages of correct responses of the two groups of students to the individual questions on hand hygiene knowledge are given in Table 1.

## 5. Attitudes to Hand Hygiene

The majority of students had poor attitudes with regard to hand hygiene. Nursing students had significantly (*P* < 0.05) better attitudes (52.1%) compared to medical students (12.9%). The percentages of correct responses of the two groups of students to the individual questions on hand hygiene attitudes are given in Table 2.

## 6. Practices of Hand Hygiene

Nursing students had significantly (*P* < 0.05) better practices (62.1%) compared to medical students (19.6%) and the difference was statistically significant (*P* < 0.05). The percentages of correct responses of the two groups of students to the individual questions on hand hygiene practices are given in Table 3.

## 7. Discussion

In our study, both study groups had moderate knowledge on hand hygiene, which was a positive finding. Feather et al. [12] studied the hand hygiene practices of 187 candidates during final MBBS OSCE (Objective Structured Clinical Examination) at The Royal London Hospital School of Medicine and Dentistry in UK and found that only 8.5% of candidates washed their hands after patient contact, although the figure rose to 18.3% when hand hygiene signs were displayed. The situation in healthcare centers of developing countries is even more unacceptable [9]. In an earlier study from Saudi Arabia [14], adherence to hand hygiene was seen in 70% of medical students, 18.8% of nurses, and 9.1% of senior medical staff, but the technique was suboptimal in all. Like most previous studies, our study showed that the overall compliance of hand hygiene by HCWs was less than 50% [15]. However, compliance with hand hygiene practice differed among different professional categories of HCWs. Compliance among the physician category was low, compared to nursing groups. Van de Mortel et al. in 2010 [16] compared the hand hygiene knowledge, beliefs, and practices between nursing and medical students. They found that the nursing students hand hygiene knowledge was significantly higher than that of medical students (*P* < 0.01) which is consistent with our study.

Our study shows the importance of improving the current training programs targeting hand hygiene practices among medical and nursing students. Hand hygiene training sessions may need to be conducted more frequently for medical students with continuous monitoring and performance feedback to encourage them to follow correct hand hygiene practices.

## Figures and Tables

**Table 1:** Comparison of knowledge in medical and nursing students on each question.

Slot number	Questions (answers)	Medical students (*n* = 98)	Nursing students (*n* = 46)	*P* value
1	Which of the following is the main route of transmission of potentially harmful germs between patients? (health care workers hands when not clean)	74 (75.6 %)	32 (76.2%)	NS
2	What is the most frequent source of germs responsible for health care associated infections? (germs already present on or within the patient)	44 (41.5%)	12 (26.6%)	0.0025

Which of the following hand hygiene actions prevents transmission of germs to the patient?				
3	Before touching a patient (yes)	90 (91.6%)	45 (97.8%)	NS
4	Immediately after risk of body fluid exposure (yes)	81 (82.4%)	39 (84.3%)	NS
5	After exposure to immediate surroundings of a patient (no)	26 (26.7%)	13 (28.4%)	NS
6	Immediately before a clean/aseptic procedure (yes)	79 (80.3%)	40 (86.7%)	NS

Which of the following hand hygiene actions prevents transmission of germs to the health care worker?				
7	After touching a patient (yes)	92 (94.2%)	46 (99.6%)	0.02
8	Immediately after a risk of body fluid exposure (yes)	86 (87.8%)	41 (90.2%)	NS
9	Immediately before a clean/aseptic procedure (no)	48 (48.9%)	28 (61.2%)	0.05
10	After exposure to the immediate surroundings of a patient (yes)	70 (71.2%)	35 (77.1%)	NS

Which of the following statements on alcohol-based hand rub and hand washing with soap and water is true?				
11	Hand rubbing is more rapid for hand cleansing than hand washing (true)	68 (69.6%)	36 (78.9%)	NS
12	Hand rubbing causes skin dryness more than hand washing (false)	30 (30.2%)	10 (20.8%)	NS
13	Hand rubbing is more effective against germs than hand washing (false)	45 (45.7%)	16 (34.2%)	0.01
14	Hand washing and hand rubbing are recommended to be performed in sequence (false)	45 (46.3%)	11 (24.2%)	NS
15	What is the minimal time needed for alcohol-based hand rub to kill most germs on your hands? (20 seconds)	38 (38.3%)	13 (27.8%)	NS

Which type of hand hygiene method is required in the following situations?				
16	Before palpation of the abdomen (rubbing)	27 (27.3%)	18 (38.8%)	0.02
17	Before giving an injection (rubbing)	25 (25.2%)	14 (31.4%)	NS
18	After emptying a bed pan (washing)	67 (68.2%)	34 (79.8%)	0.02
19	After removing examination gloves (rubbing/washing)	64 (65.6%)	36 (78.7%)	NS
20	After making a patient's bed (rubbing)	30 (30.9%)	6 (12.6%)	0.0005
21	After visible exposure to blood (washing)	46 (46.7%)	27 (57.9%)	0.03

Which of the following should be avoided, as associated with increased likelihood of colonization of hands with harmful germs?				
22	Wearing jewellery (yes)	76 (77.7%)	44 (96.6%)	0.0001
23	Damaged skin (yes)	93 (95.3%)	43 (93.9%)	NS
24	Artificial fingernails (yes)	79 (80.9%)	41 (90.1%)	0.04
25	Regular use of a hand cream (no)	54 (54.8%)	31 (67.4%)	NS

*Z* test. *P* < 0.05 (significant), *P* < 0.001 (highly significant), and NS (not significant).

**Table 2:** Comparison of hand hygiene practice among medical and nursing students.

Slot number	Statement	Medical students (*n* = 98)	Nursing students (*n* = 46)	*P* value
1	I adhere to correct hand hygiene practices at all times	21 (21.4%)	28 (61.8%)	<0.001
2	I have sufficient knowledge about hand hygiene	35 (35.3%)	34 (74.4%)	<0.001
3	Sometimes I have more important things to do than hand hygiene	20 (20.5%)	16 (35.2%)	0.004
4	Emergencies and other priorities make hygiene more difficult at times	74 (7.6%)	22 (4.7%)	NS
5	Wearing gloves reduces the need for hand hygiene	25 (25.8%)	18 (38.6%)	0.01
6	I feel frustrated when others omit hand hygiene	27 (27.6%)	25 (54.5%)	<0.001
7	I am reluctant to ask others to engage in hand hygiene	21 (21.2%)	8 (16.4%)	NS
8	Newly qualified staff has not been properly instructed in hand hygiene in their training	26 (26.6%)	23 (49.8%)	<0.001
9	I feel guilty if I omit hand hygiene	39 (39.4%)	32 (68.9%)	<0.001
10	Adhering to hand hygiene practices is easy in the current setup	27 (27.2%)	21 (46.1%)	0.008

*Z* test. *P* < 0.05 (significant), *P* < 0.001 (highly significant), and NS (not significant).

**Table 3:** Comparison of the correct responses to hand hygiene practices of medical and nursing students.

Slot number	Statement	Medical students (*n* = 98)	Nursing students (*n* = 46)	*P* value
1	Sometimes I miss out hand hygiene simply because I forget it	16 (16.2%)	21 (46.1%)	<0.001
2	Hand hygiene is an essential part of my role	46 (46.7%)	38 (83.6%)	<0.001
3	The frequency of hand hygiene required makes it difficult for me to carry it out as often as necessary	63 (6.4%)	13 (27.7%)	<0.001
4	Infection prevention team have a positive influence on my hand hygiene	20 (20.7%)	25 (54.8%)	<0.001
5	Infection prevention notice boards remind me to do hand hygiene	26 (26.5%)	24 (52.9%)	<0.001
6	It is difficult for me to attend hand hygiene courses due to time pressure	11 (11.4%)	14 (30.3%)	<0.001

*Z* test. *P* < 0.05 (significant), *P* < 0.001 (highly significant), and NS (not significant).
